# Mechanical Properties and In Vitro Biocompatibility of Hybrid Polymer-HA/BAG Ceramic Dental Materials

**DOI:** 10.3390/polym14183774

**Published:** 2022-09-09

**Authors:** Yuanyuan Chen, Cheng Sun, Jinfang Cao, Yuanyuan Wu, Bencang Cui, Jianfeng Ma, Huining Wang

**Affiliations:** 1 School and Hospital of Stomatology, Wenzhou Medical University, Wenzhou 325027, China; 2 Foshan (Southern China) Institute for New Materials, Foshan 528247, China; 3 State Key Laboratory of New Ceramics and Fine Processing, School of Materials Science and Engineering, Tsinghua University, Beijing 100084, China

**Keywords:** hybrid polymer–ceramic, hydroxyapatite, bioactive glass, mechanical properties, human gingival fibroblasts

## Abstract

The aim of this study is to prepare hybrid polymer–ceramic dental materials for chairside computer-aided design/computer-aided manufacturing (CAD/CAM) applications. The hybrid polymer–ceramic materials were fabricated via infiltrating polymerizable monomer mixtures into sintered hydroxyapatite/bioactive glass (HA/BAG) ceramic blocks and thermo-curing. The microstructure was observed by scanning electron microscopy and an energy-dispersive spectrometer. The phase structure was analyzed by X-ray diffraction. The composition ratio was analyzed by a thermogravimetric analyzer. The hardness was measured by a Vickers hardness tester. The flexural strength, flexural modulus, and compressive strength were measured and calculated by a universal testing machine. The growth of human gingival fibroblasts was evaluated by a 3-(4,5-dimethylthiazol-2-yl)-2,5-diphenyl tetrazolium bromide (MTT) colorimetric assay and immunofluorescence staining. The results showed that the sintering temperature and BAG content affected the mechanical properties of the hybrid polymer–ceramic materials. The X-ray diffraction analysis showed that high-temperature sintering promoted the partial conversion of HA to β-tricalcium phosphate. The values of the hardness, flexural strength, flexural modulus, and compressive strength of all the hybrid polymer–ceramic materials were 0.89–3.51 GPa, 57.61–118.05 MPa, 20.26–39.77 GPa, and 60.36–390.46 MPa, respectively. The mechanical properties of the hybrid polymer–ceramic materials were similar to natural teeth. As a trade-off between flexural strength and hardness, hybrid polymer–ceramic material with 20 wt.% BAG sintered at 1000 °C was the best material. In vitro experiments confirmed the biocompatibility of the hybrid polymer–ceramic material. Therefore, the hybrid polymer–ceramic material is expected to become a new type of dental restoration material.

## 1. Introduction

Teeth defects, dentition defects, and missing teeth caused by caries, periodontal disease, and trauma have promoted the development of dentally restorative materials. In recent years, chairside computer-aided design/computer-aided manufacturing (CAD/CAM) has become an increasingly popular technology for personalized restorations. The machinable dental materials should possess good machinability, high strength, high cutting efficiency, and the ability to be easily polished [1].

Hydroxyapatite (HA) ceramics, biphasic calcium phosphate (BCP) ceramics, and bioactive glass (BAG) ceramics have been widely used in the biomedical field. BCP is composed of HA and β-tricalcium phosphate (β-TCP). BCP ceramics have proven to be adequately biocompatible, bioactive, and osteoconductive materials [2]. Compared with pure HA ceramics and pure β-TCP ceramics, BCP ceramics have more appropriate degradation/redeposition rates, stronger mechanical properties, and osteoinductive effects [3,4,5,6,7]. BAG has good biomineralization and forms an apatite layer in simulated body fluids [8]. It also promotes the proliferation of osteoblast-like cells and the differentiation of keratinocytes [9,10]. Dental ceramics with a small amount of BAG support the proliferation of human gingival fibroblasts. The attachment of connective tissue also acts as a barrier against oral bacteria, extending the retention time of the restoration [11]. In addition, the bioactivity of HA/BAG ceramics with a high BAG addition is significantly superior to that of pure HA ceramics [12]. However, HA/BAG ceramics have a high brittleness and a low transparency. They were mainly used to repair damaged and defective bone tissue in the past [13,14,15]. They were not suitable for dental crown restoration due to aesthetic reasons and their poor mechanical properties.

Hybrid polymer–ceramic materials are a new type of CAD/CAM machinable material. They combine the advantages of both polymer and ceramics. Owing to their unique dual network structure, the stiffness, damage tolerance, flexibility, and fracture toughness of hybrid polymer–ceramic materials are superior to porous ceramics [16]. Compared with high elastic modulus repair materials such as zirconia and alumina, hybrid polymer–ceramic materials, with a low elastic modulus, present a lower stress concentration on a structure and on the restoration intaglio surface [17].

Few previous experiments have been conducted on hybrid polymer–HA/BAG ceramic materials. Thus, the aim of this study is to fabricate hybrid polymer–ceramic composites composed of polymer and HA/BAG. The mechanical and biocompatible properties of the obtained composites were characterized to demonstrate their applicability and superiority in dental restorative materials.

## 2. Materials and Methods

### 2.1. Materials

The bioactive glass (BAG, Guangzhou Corgen Material Technology Co., Ltd., Guangzhou, China) used was composed of 59 mol% SiO_2_, 36 mol% CaO and 5 mol% P_2_O_5_. Hydroxyapatite (HA, Shanghai Yuanye Bio-technology Co., Ltd., Shanghai, China) was the main raw material for ceramics. Deionized water was obtained from a deionized water purifier (Smart-Q15, Shanghai HHitech Instrument Co., Ltd., Shanghai, China). Sodium carboxymethyl cellulose (CMC-Na, Shanghai Aladdin Biochemical Technology Co., Ltd., Shanghai, China) and polyethylene glycol (PEG, Shanghai Titan Scientific Co., Ltd., Shanghai, China) were used as binder and dispersant, respectively. Bisphenol-A-glycidyl dimethacrylate (Bis-GMA, Merck, Darmstadt, Germany), triethylene glycol dimethacrylate (TEGDMA, Shanghai Aladdin Biochemical Technology Co., Ltd., Shanghai, China), and dibenzoyl peroxide (BPO, J & K Scientific LTD, St. Louis, MO, USA) were components of the polymerizable monomer mixtures.

### 2.2. Preparation of Hybrid Polymer–Ceramic Materials

Slurries were prepared by mixing deionized water with HA (5–20 µm), *n* wt.% (*n* = 10, 15, 20 and 25) of BAG (5 µm), 2.5 wt.% CMC-Na, and 2.5 wt.% PEG. The solid–liquid ratio was 1:3.5. The preparation of slurries referred to the articles of Tancred [18] and Chen [19]. Pure BAG and pure HA were also added with binders and dispersants as control groups, named 100 wt.% BAG and 0 wt.% BAG. The slurries were ball-milled with zirconia balls for 3.5 h at 200 r/min, passed through a 100-mesh sieve, and dried in a drying oven at 65 °C for 12 h. The dried powder was ground in a mortar. Molding processes included first compression molding (2 MPa, 30 s) and then cold isostatic pressing (250 MPa, 1 min). The green bodies were sintered at 1000 °C, 1100 °C, 1200 °C, and 1300 °C, with a heating rate of 2 °C/min and preservation time of 3 h. A total of 24 different ceramic blocks were prepared.

**Figure 1:**
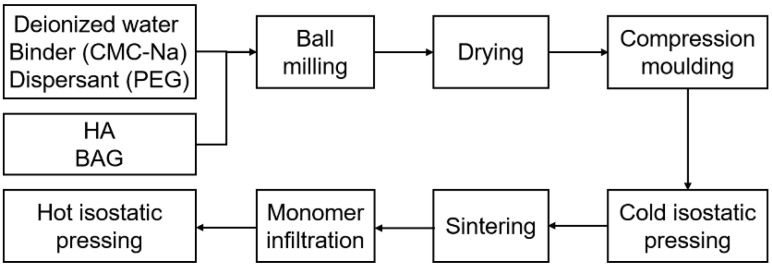
The processing steps for the preparation of the hybrid polymer–ceramic materials.

The polymerizable monomer mixtures were formulated with Bis-GMA and TEGDMA in a mass ratio of 1:1. A 1 wt.% BPO was used as the thermal initiator. They were stirred with a magnetic stirrer for 24 h until transforming into a homogeneous and low viscous liquid. The ceramic blocks were immersed in the monomer mixtures and placed in a vacuum-drying oven with a vacuum degree of −0.1 MPa for 3 days. Compared with normal atmospheric pressure, vacuum capillary action accelerated the complete infiltration of the monomer mixtures into the ceramic blocks. Finally, they were cured by hot isostatic pressing at 100 °C under a pressure of 150 MPa. Under the same conditions, the monomer mixtures were cured into pure resins as a control group. The process flow is shown in Figure 1.

Cured hybrid polymer–ceramic blocks were cut to the appropriate dimensions by a cutting machine (SYJ-400, Shenyang Kejing Auto-instrument Co., Ltd., Shenyang, China) equipped with an electroplated diamond saw blade. The spindle rotation speed of the cutting machine was 1500 rpm, and the feed rate was 2 mm/min. The test specimens were polished with 500, 2000, and 4000 grit silicon carbide (SiC) abrasive papers in turn. Finally, the dimensions of the cubic specimens were (25 ± 2) mm × (6 ± 0.1) mm × (3 ± 0.1) mm (*n* = 2), (25 ± 2) mm × (2 ± 0.1) mm × (2 ± 0.1) mm (*n* = 10) and (3 ± 0.1) mm × (3 ± 0.1) mm × (5 ± 0.1) mm (*n* = 10). After their cutting and polishing with 500 and 2000 grit SiC abrasive paper, cured pure resins, ceramic blocks, and cured hybrid polymer–ceramic blocks used in vitro experiments were (3 ± 0.1) mm × (3 ± 0.1) mm × (1 ± 0.1) mm (*n* = 12).

### 2.3. Microstructure and Phase Characterization

The cross-section of the specimens after cutting was treated with gold spraying. The microstructure was observed by a field emission scanning electron microscope (FESEM, Apreo 2S, Thermo Fisher Scientific, St. Louis, MO, USA). Elemental species and content analysis were differentiated by an energy dispersive spectrometer (Ultim MaX65, Oxford, Wycombe, UK). Crystal structure analysis was performed using an X-ray diffractometer (D8 Advance, Bruker, Karlsruhe, Germany).

### 2.4. Thermogravimetric Analysis

Proportion of polymers and ceramics in hybrid polymer–ceramic composites was analyzed by a thermogravimetric analyzer (STA449F5, Netzsch, Selb, Germany) under O_2_ atmosphere at heating rate of 10 °C/min.

### 2.5. Surface Roughness

The surface roughness of (3 ± 0.1) mm × (3 ± 0.1) mm × (1 ± 0.1) mm specimens for in vitro experiments was tested by a 3D digital microscope (DSX1000, Olympus Corporation, Tokyo, Japan). Arithmetic mean deviation (Pa) and root mean square deviation (Pq) were calculated by the system.

### 2.6. Porosity and Shrinkage

The porosity of the sintered ceramics was measured by the Archimedes drainage method. The shrinkage rate was calculated by using the volume of the ceramics before and after sintering. The ceramic specimens were not cut and polished. The dimensions of specimens for both tests were (25 ± 2) mm × (10 ± 2) mm × (10 ± 2) mm (*n* = 5). Porosity was calculated by Equation (1):

(1) $$P=\frac{m_{3}-m_{1}}{m_{3}-m_{2}}\times 100\%$$

where m_1_ is the mass of the dry ceramic blocks in the air; m_2_ is the mass of saturated ceramic blocks in the water; m_3_ is the mass of saturated ceramic blocks in the air.

### 2.7. Hardness

The Vickers hardness was tested by a Vickers hardness tester (VH1150, Wilson, MO, USA). The dimensions of specimens were (25 ± 2) mm × (6 ± 0.1) mm × (3 ± 0.1) mm (*n* = 2). A total of 10 points were randomly selected to apply the load on each cubic specimen surface. The mechanical properties of natural human teeth (Table 1) were used as a reference.

### 2.8. Flexural Strength and Flexural Modulus

The three-point bending test was performed on a universal testing machine (AGS, Shimadzu, Tokyo, Japan). According to ISO 10477: 2018 and ISO 4049: 2019 standards of dental restorative materials, 10 cubic specimens were prepared for each of the groups with the size of (25 ± 2) mm × (2 ± 0.1) mm × (2 ± 0.1) mm. The following formulas were used: for flexural strength (Equation (2))

(2) $$\sigma_{f}=\frac{3\mathrm{Fl}}{{2\mathrm{bh}}^{2}}$$

for flexural modulus (Equation (3))

(3) $$E_{f}=(\frac{F}{m})\frac{l^{3}}{{4\mathrm{bh}}^{3}}$$

where F is the maximum load; (F/m) is the slope of the force-displacement curve; l is the distance between the supports (l = 20 mm); and b and h are the width and height, respectively, at the center of the specimen measured instantly prior to testing. The loading speed was 1 mm/min.

### 2.9. Compressive Strength

Tests were carried out on a universal testing machine (AGS, Shimadzu, Tokyo, Japan). 10 cubic specimens were prepared for each group with the size of (3 ± 0.1) mm × (3 ± 0.1) mm × (5 ± 0.1) mm. A (3 ± 0.1) mm × (3 ± 0.1) mm surface area was loaded with pressure. The compressive strength of the specimens was evaluated according to Equation (4):

(4) $$\sigma =\frac{F}{S}$$

where F is the maximum load and S is the force area. The loading speed was 0.5mm/min.

### 2.10. In Vitro Cell Biocompatibility Measurements

To examine the cell viability of human gingival fibroblasts (HGFs, BT200003, Wuhan Biotower Biotechnology Co., Ltd., Wuhan, China) on the hybrid polymer–ceramic materials, direct contact experiment was performed by a 3-(4,5-dimethylthiazol-2-yl)-2,5-diphenyl tetrazolium bromide (MTT, HY-15924, MCE, Concord, CA, USA) colorimetric assay. HGFs were seeded in cell culture medium. The cell culture medium contained Dulbecco’s modified Eagle medium (DMEM, PM150210, Procell Life Science & Technology Co., Ltd., Beijing, China), 10% fetal bovine serum (FBS, 111323, Shanghai Excell Biological Technology Co., Ltd., Shanghai, China), and 1% penicillin-streptomycin (PB180120, Procell Life Science & Technology Co., Ltd., China). HGFs were cultured in an incubator with 5% CO_2_ at 37 °C. Cells were passaged using 0.25% trypsin (PB180225, Procell Life Science & Technology Co., Ltd., China). HGFs were seeded at 5 × 10^3^ cells/well in 96-well cell culture plates containing the cubic specimens. The specimens (cured pure resins, 20 wt.% BAG ceramics sintered at 1000 °C, and hybrid polymer–ceramic materials of 20 wt.% BAG sintered at 1000 °C) were cleaned with ethanol and sterilized under ultraviolet light for 30 min. Their dimensions were (3 ± 0.1) mm × (3 ± 0.1) mm × (1 ± 0.1) mm (*n* = 12). The same culture medium without the specimen was used as a negative control. The growth status of cells was observed after 48 h and 72 h. After 72 h cell culture, 10 mL of MTT was added to each well and was incubated at 37 °C for 4 h. After that, the medium was removed. A total of 150 μL of DMSO was added and shaken for 10 min. The optical density (OD) was measured at a wavelength of 570 nm with a microplate reader (C22.2NO.1010.1, BioTek Instruments, Winooski, VT, USA). Results were expressed as a percentage of the negative control values.

HGFs grown for 72 h were fixed with 4% paraformaldehyde (8,009,6618, China National Medicines Co., Ltd., Beijing, China) for 15 min. Cells were dual stained with phalloidin (40734ES75, Yeasen Biotechnology (Shanghai) Co., Ltd., Shanghai, China) and 4,6-Diamidino-2-phenyindole dilactate (DAPI, C1002, Shanghai Beyotime Biotechnology Co., Ltd., Shanghai, China). A solution containing anti-fluorescence quencher (0100-01, Southernbiotech, Birmingham, AB, USA) was added dropwise. The extension of labeled cells was observed by laser confocal microscopy (FV3000, Olympus Corporation, Tokyo, Japan).

### 2.11. Statistical Analysis

The results were statistically analyzed by SPSS software (SPSS 26.0, IBM Corp., Armonk, NY, USA) and compared using One-Way ANOVA and Tukey’s honestly significant difference. Normality test using Shapiro–Wilk test. The significance level was 0.05.

## 3. Results

### 3.1. Macromorphology and Microstructure

**Figure 2:**
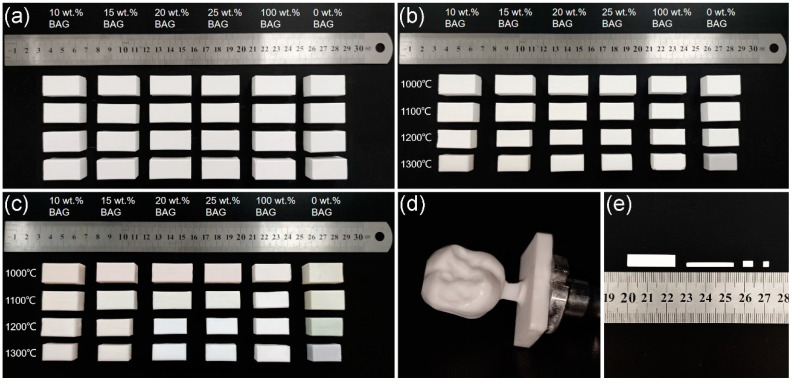
Macromorphologies of the specimens: (**a**) green bodies after cold isostatic pressing, (**b**) ceramic blocks after high-temperature sintering, (**c**) specimens after monomer mixtures’ infiltration and curing, (**d**) hybrid polymer–ceramic material dental crown with 20 wt.% BAG sintered at 1000 °C, and (**e**) cubic test specimens after cutting.

Figure 2a shows the green bodies after cold isostatic pressing. Figure 2b shows the sintered ceramics. Figure 2c shows the final specimens. Of all the observed specimen surfaces, the color changes of the pure HA (i.e., 0 wt.% BAG) groups were the most obvious. With the increase in the temperature, it became light gray, greyish-green, and gray, in turn. The color of the HA/BAG groups sintered at 1000 °C was light pink. The HA/BAG groups sintered at 1100 °C, the 10 wt.% and 15 wt.% BAG sintered at 1200 °C, and the 10 wt.% and 15 wt.% BAG sintered at 1300 °C were light yellow. The others were milky white. When the ceramics were sintered at 1000 °C and 1100 °C, uniform color distribution was observed over the cross-section of the corresponding composites. However, the periphery of the specimens at 1200 °C was light yellow and the interior was milky white. The specimens at 1300 °C were milky white on both the periphery and the interior.

Comparing Figure 2a,b, the shrinkage of the specimens significantly increased with the increase in the sintering temperature. In Figure 2d, the crown restoration was carved from a hybrid polymer–ceramic block by a denture-processing machine (SD-5SH, Shanghai Shendiao, Shanghai, China). The milled edges were smooth, and no cracks or disintegration were observed. In Figure 2e, the test specimens of different sizes were cut from hybrid polymer–ceramic blocks and then polished.

**Figure 3:**
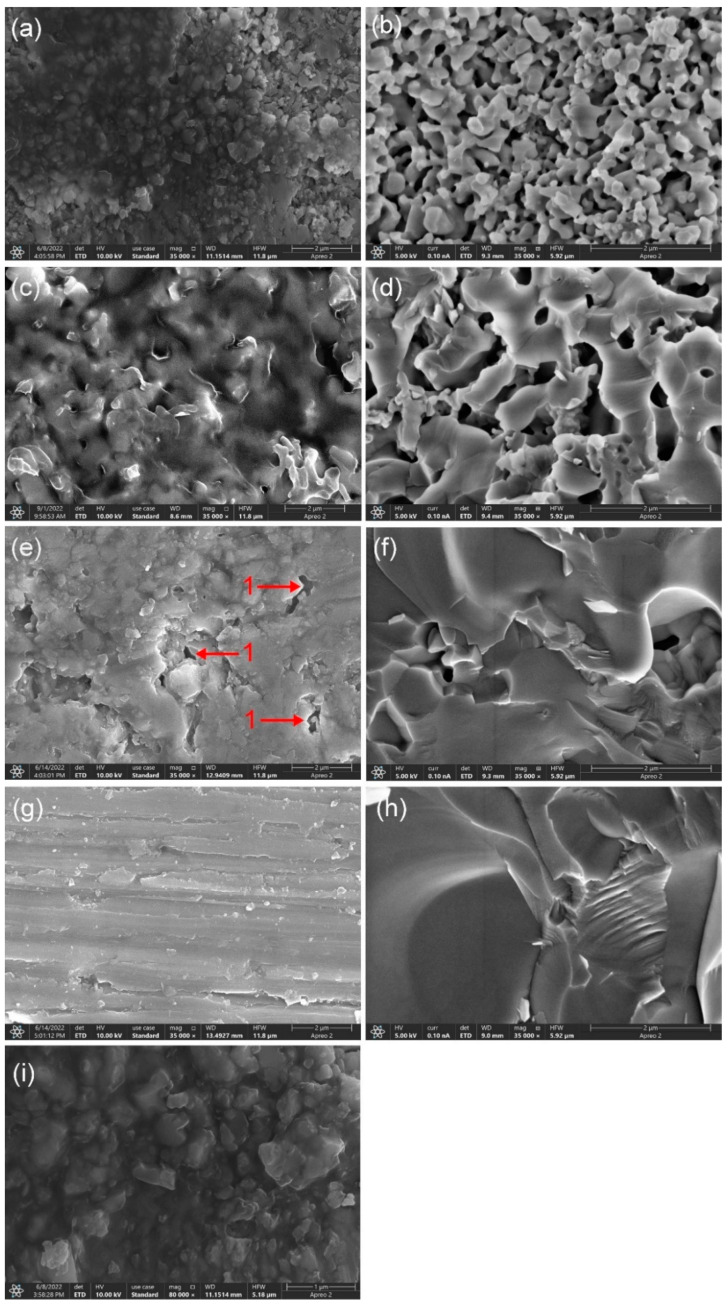
FESEM images of fracture surfaces of specimens with different sintering temperatures: (**a**,**b**,**i**) 1000 °C; (**c**,**d**) 1100 °C; (**e**,**f**) 1200 °C; (**g**,**h**) 1300 °C; (**a**,**c**,**e**,**g**,**i**) specimens after monomer mixtures’ infiltration and curing; (**b**,**d**,**f**,**h**) ceramics; arrow 1 in (**e**) closed pores; (**i**) enlarged view of (**a**).

Figure 3 shows the microstructure of the fracture surfaces of the 20 wt.% BAG specimens with different sintering temperatures. The density of the porous ceramic was found to increase with the increasing of sintering temperature (Figure 3b,d,f,h). Finally, a dense ceramic was obtained at 1300 °C. Interconnected micropores and complete infiltration were observed in the specimens sintered at 1000 °C and 1100 °C (Figure 3a,c). Comparing Figure 3a,b and Figure 3c,d, the dark area represents the polymer phase and the bright area represents the ceramic phase. Figure 3i at a high magnification shows that the boundary between the polymer phase and the ceramic phase is blurred. A double network structure was formed by the close interconnection of the two phases. The specimen sintered at 1200 °C had more interconnected micropores in the periphery and fewer closed pores in the interior. The polymer phase was rarely observed in the interior. The specimens sintered at 1300 °C were relatively dense. The closed pores were smaller than the micropores of the specimens sintered at 1200 °C. The polymer phase was not observed whatsoever.

### 3.2. Energy Spectrum Analysis

**Figure 4:**
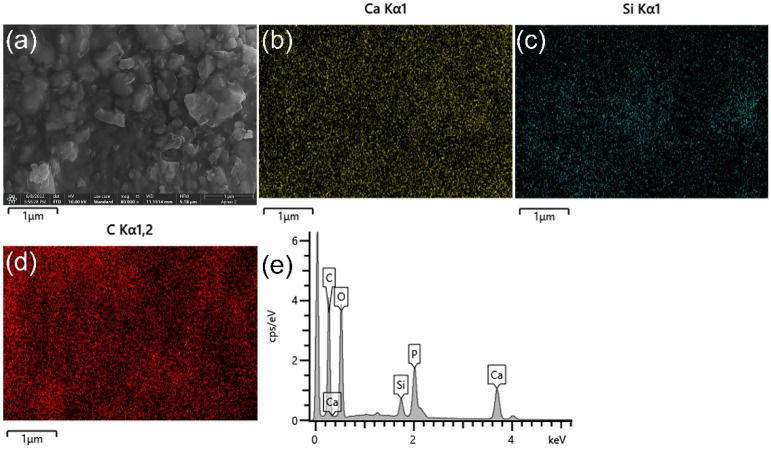
Energy spectrum of the hybrid polymer–ceramic material fracture surfaces of 20 wt.% BAG sintered at 1000 °C: (**a**) secondary electron image of hybrid polymer–ceramic material; (**b**–**d**) the distribution diagrams of Ca, Si, and C in (**a**), respectively; (**e**) the energy spectrum of (**a**).

Figure 4 shows the energy spectrum of the hybrid polymer-ceramic material with 20 wt.% BAG sintered at 1000 °C. Si represents the BAG and C represents the polymer. Ca represents the ceramic phase, which is contained in both the HA and BAG. It can be seen from Figure 4 that the distribution of the HA, BAG, and polymer is relatively uniform. In addition, the energy spectrum shows that the C content of 20 wt.% BAG groups sintered at 1000–1300 °C are 32.85 wt.%, 28.09 wt.%, 19.08 wt.%, and 15.75 wt.%. No polymer phases were found in the interior of the ceramics sintered at 1200 °C and in the ceramics sintered at 1300 °C. Therefore, the specimens at 1200 °C and 1300 °C are not hybrid polymer–ceramic materials.

### 3.3. X-ray Diffraction (XRD) Analysis

**Figure 5:**
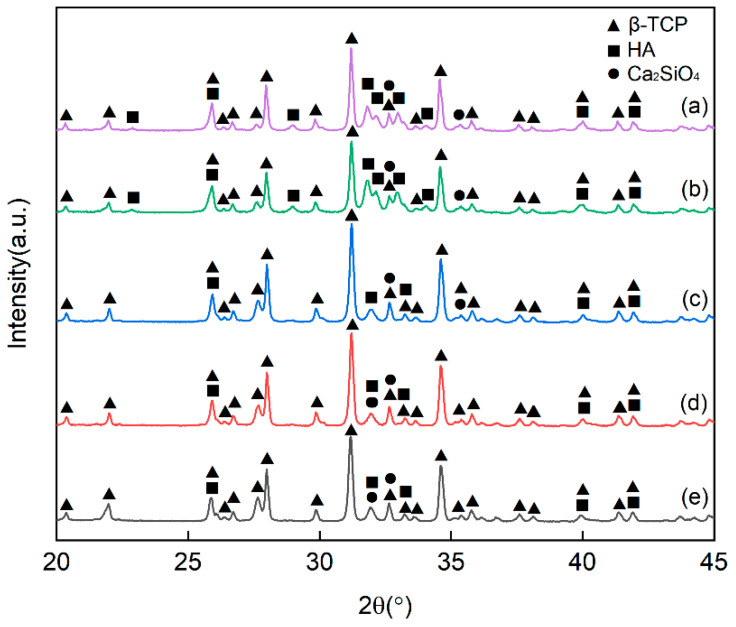
XRD patterns of ceramics with different sintering temperatures and different BAG contents: (**a**)1000 °C and 10 wt.% BAG; (**b**)1000 °C and 20 wt.% BAG; (**c**)1100 °C and 20 wt.% BAG; (**d**)1200 °C and 20 wt.% BAG; (**e**)1300 °C and 20 wt.% BAG.

When the sintering temperature increased, the diffraction peak of HA gradually weakened, the content of HA decreased, and the content of tricalcium phosphate (TCP) increased (Figure 5c–e). There are three polymorphic forms of TCP. The β phase appears below 1180 °C. The α phase appears at 1180–1400 °C. If the α phase is cooled slowly, it will change to the β phase. The α’ phase appears above 1470 °C [24,25]. Comparing the three allotropes, β-TCP has good biocompatibility and mechanical strength. Its degradation rate is lower than that of α-TCP [26]. The XRD patterns of the ceramics were compared with the β-TCP standard card. The characteristic peaks and the sub-strength peaks of the two were consistent. Characteristic peaks of α-TCP phase were not found, indicating that only β-TCP was generated under this experimental condition. In addition, a new phase, Ca2SiO4, was also generated.

Figure 5a,b are ceramics with the same sintering temperature (1000 °C). When the BAG content increased from 10 wt.% to 20 wt.%, the degree of conversion from HA to β-TCP decreased. This is similar to the experimental result of Tancred [18].

### 3.4. Thermogravimetric Analysis

**Figure 6:**
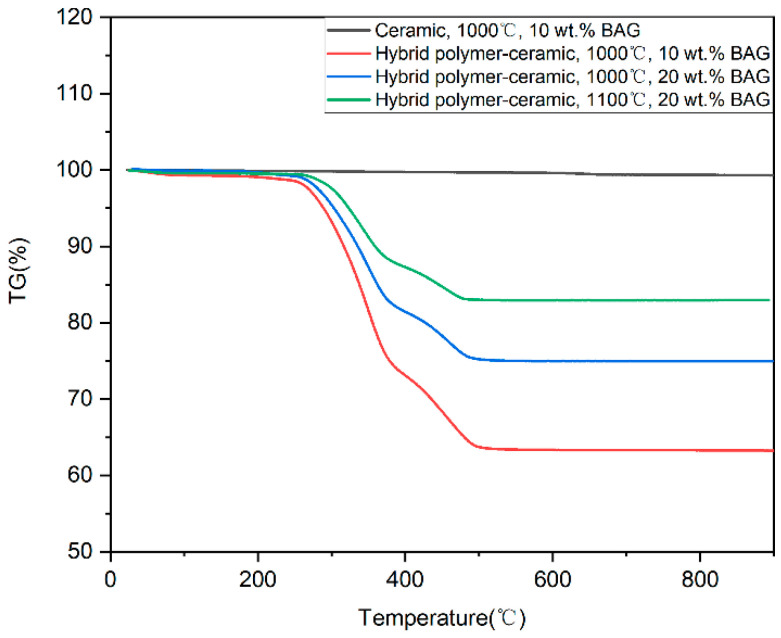
TG curves of HA/BAG ceramic and hybrid polymer–ceramic materials.

Figure 6 displays the thermogravimetric (TG) curves of the HA/BAG ceramic and hybrid polymer–ceramic materials with different sintering temperatures and different BAG contents. The quality of the HA/BAG ceramic with 10 wt.% BAG sintered at 1000 °C hardly changed with the temperature. Compared with the ceramic, the hybrid polymer–ceramic materials’ curves had obvious steps and a substantial decline in quality. The cured polymers in the hybrid polymer–ceramic materials underwent a thermal cracking reaction with the increase in the temperature. In the hybrid polymer–ceramic material with 10 wt.% BAG sintered at 1000 °C, bound water accounted for 1.21%, ceramic for 63.18%, and polymer for 35.61%. In the hybrid polymer–ceramic material with 20 wt.% BAG sintered at 1000 °C, ceramic accounted for 74.97% and polymer for 17.9%. In the hybrid polymer–ceramic material with 20 wt.% BAG sintered at 1100 °C, ceramic accounted for 83.96% and polymer for 12.38%. This illustrated that with the increase in the BAG content or sintering temperatures, the proportion of ceramics increases and the proportion of polymers decreases in the hybrid polymer–ceramic materials.

### 3.5. Surface Roughness

**Figure 7:**
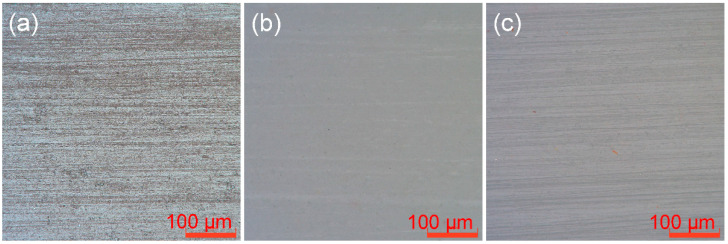
Surface microstructure of different materials: (**a**) cured pure resin; (**b**) ceramic; (**c**) hybrid polymer–ceramic material.

The dimensions of the three materials after polishing with 500 and 2000 grit SiC abrasive papers were (3 ± 0.1) mm × (3 ± 0.1) mm × (1 ± 0.1) mm (*n* = 12). The materials were cured pure resin, ceramic with 20 wt.% BAG sintered at 1000 °C, and hybrid polymer–ceramic material with 20 wt.% BAG sintered at 1000 °C (Figure 7). Typical grooves and damage are visible on the surface of the materials. The grain direction is in the polishing direction. It can be seen from Table 2 that the order of the surface roughness of the different materials prepared by the same cutting machine and the same abrasive papers is as follows: cured pure resin > hybrid polymer–ceramic material > ceramic.

### 3.6. Shrinkage and Porosity

**Figure 8:**
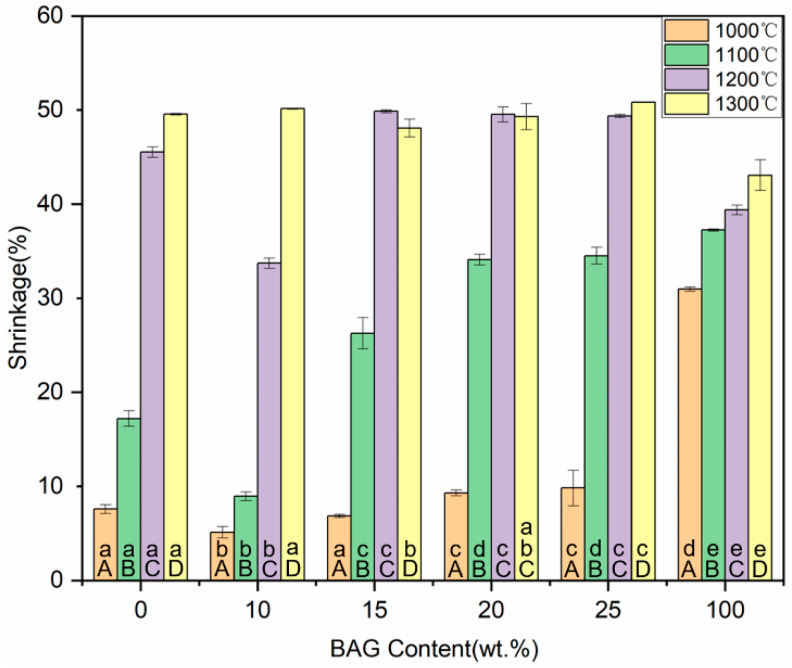
Shrinkage of ceramics with different sintering temperatures and different BAG contents. ^a^ the same lowercase letters indicate that there was no significant difference between different BAG contents at the same sintering temperature (*p* = 0.05). ^A^ the same uppercase letters indicate that there was no significant difference between different sintering temperatures with the same BAG content (*p* = 0.05). The data were significantly drawn from a normally distributed population at the 0.05 level.

**Figure 9:**
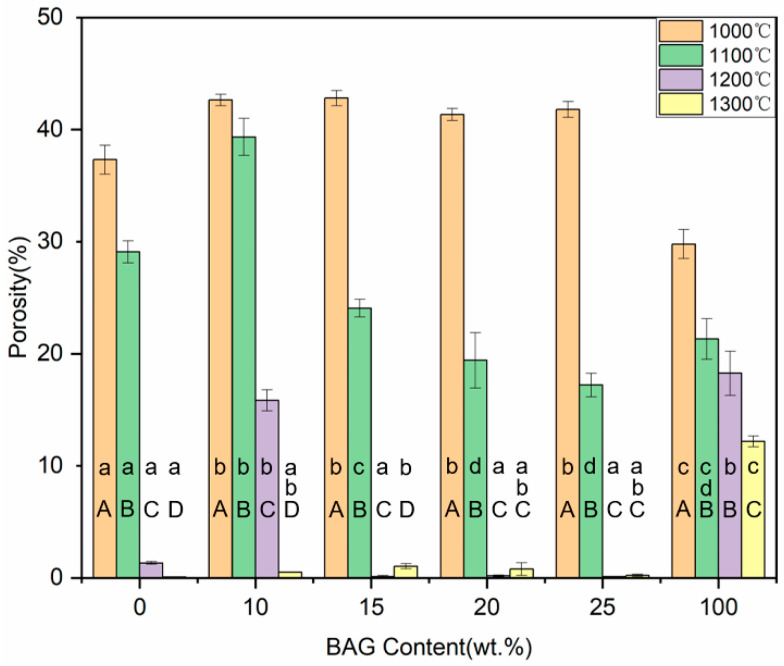
Porosity of ceramics with different sintering temperatures and different BAG contents. ^a^ the same lowercase letters indicate that there was no significant difference between different BAG contents at the same sintering temperature (*p* = 0.05). ^A^ the same uppercase letters indicate that there was no significant difference between different sintering temperatures with the same BAG content (*p* = 0.05). The data were significantly drawn from a normally distributed population at the 0.05 level.

Figure 8 and Figure 9 show the shrinkage and porosity of the ceramics with different sintering temperatures and different BAG contents. With the increase in the sintering temperature or BAG content, the shrinkage gradually increased, and the porosity gradually decreased. The 0 wt.%, 15 wt.%, 20 wt.%, and 25 wt.% BAG ceramics sintered at 1200 °C, and the 0–25 wt.% BAG ceramics sintered at 1300 °C, had a high shrinkage and extremely low porosity, which means that the monomer mixtures could not penetrate the ceramics. Combined with the above studies, the specimens sintered at 1000 °C and 1100 °C, 10 wt.%, and 100 wt.% BAG sintered at 1200 °C were hybrid polymer–ceramic materials. Therefore, the shrinkage of the HA/BAG ceramic areas of the hybrid polymer–ceramic materials was 5.12–33.71%, and the porosity was 15.84–42.82%. The HA/BAG ceramic with the highest porosity was the 15 wt.% BAG sintered at 1000 °C, and the lowest was the 10 wt.% BAG sintered at 1200 °C.

### 3.7. Hardness

**Figure 10:**
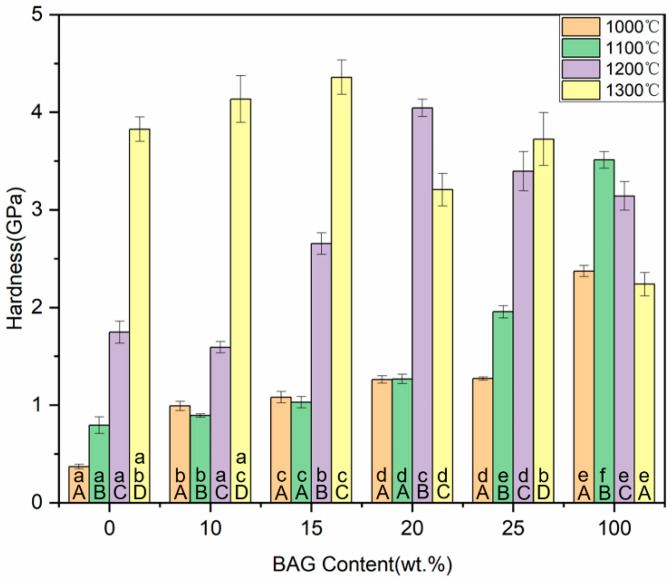
Hardness of specimens with different sintering temperatures and different BAG content .^a^ the same lowercase letters indicate that there was no significant difference between different BAG contents at the same sintering temperature (*p* = 0.05). ^A^ the same uppercase letters indicate that there was no significant difference between different sintering temperatures with the same BAG content (*p* = 0.05). The data were significantly drawn from a normally distributed population at the 0.05 level.

The hardness of the hybrid polymer–ceramic materials generally increased with the increase in the sintering temperature or BAG content (Figure 10). The uninfiltrated HA/BAG ceramics sintered at 1200 °C and 1300 °C exhibited a higher flexural strength and flexural modulus compared to the other groups related to the high density of the ceramics. The hardness of the hybrid polymer–ceramic material for 10–25 wt.% HA/BAG groups ranged from 0.89 to 1.96 GPa, and the hardest was found for the 25 wt.% BAG at 1100 °C. In the control groups, the hardness of the 100 wt.% BAG sintered at 1100 °C was the greatest (3.51 ± 0.09 GPa).

### 3.8. Flexural Strength and Flexural Modulus

**Figure 11:**
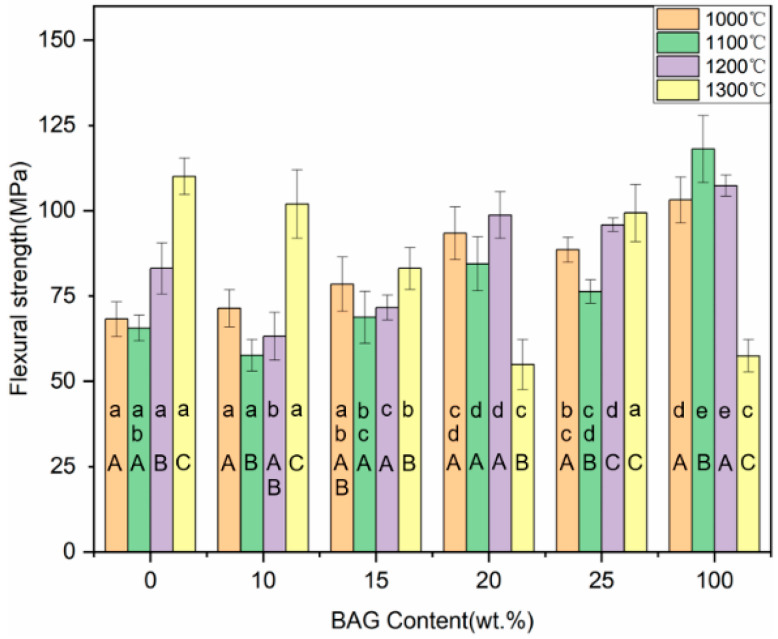
Flexural strength of specimens with different sintering temperatures and different BAG contents. ^a^ the same lowercase letters indicate that there was no significant difference between different BAG contents at the same sintering temperature (*p* = 0.05). ^A^ the same uppercase letters indicate that there was no significant difference between different sintering temperatures with the same BAG content (*p* = 0.05). The data were significantly drawn from a normally distributed population at the 0.05 level.

**Figure 12:**
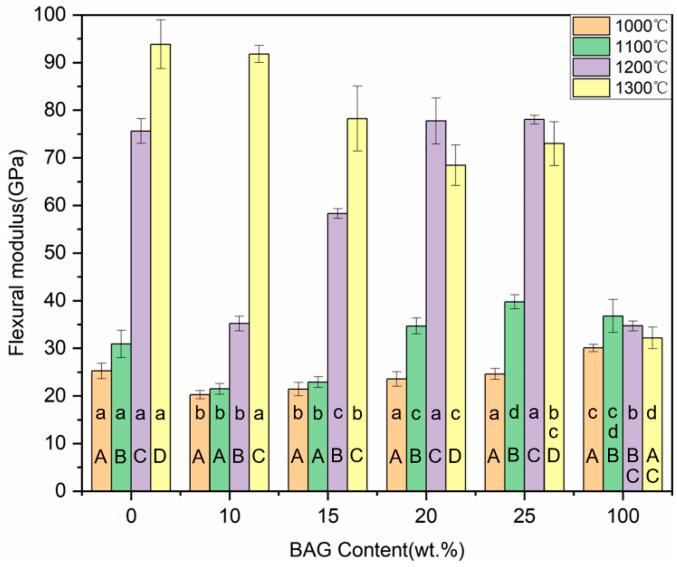
Flexural modulus of specimens with different sintering temperatures and different BAG contents. ^a^ the same lowercase letters indicate that there was no significant difference between different BAG contents at the same sintering temperature (*p* = 0.05). ^A^ the same uppercase letters indicate that there was no significant difference between different sintering temperatures with the same BAG content (*p* = 0.05). The data were significantly drawn from a normally distributed population at the 0.05 level.

The flexural strength of the hybrid polymer–ceramic materials for 10–25 wt.% BAG groups ranged from 57.61 to 93.38 MPa (Figure 11). In the control groups, the hybrid polymer–ceramic material with 100 wt.% BAG sintered at 1100 °C had the largest flexural strength (118.05 ± 9.84 MPa). With the increase in the BAG content, the flexural strength of the hybrid polymer–ceramic materials increased at first and then decreased, reaching a maximum when the BAG content was 20 wt.%. With the increase in the sintering temperature (1000 °C→1100 °C), the flexural strength of the hybrid polymer–ceramic materials decreased. As shown in Figure 12, the flexural moduli of all the hybrid polymer–ceramic materials ranged from 20.26 to 39.77 GPa. The hybrid polymer–ceramic material with 25 wt.% BAG sintered at 1100 °C had the largest flexural modulus. The flexural strength and flexural modulus of the uninfiltrated HA/BAG ceramics at 1200 °C and 1300 °C was high. This was related to the high density of the ceramics.

### 3.9. Compressive Strength

**Figure 13:**
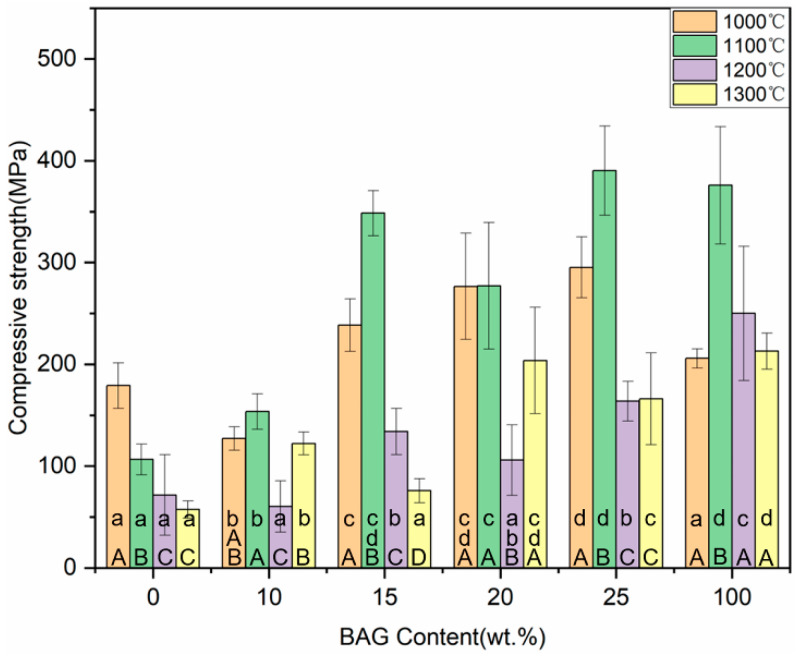
Compressive strength of specimens with different sintering temperatures and different BAG contents. ^a^ the same lowercase letters indicate that there was no significant difference between different BAG contents at the same sintering temperature (*p* = 0.05). ^A^ the same uppercase letters indicate that there was no significant difference between different sintering temperatures with the same BAG content (*p* = 0.05). The data were significantly drawn from a normally distributed population at the 0.05 level.

The posterior tooth crown has an extremely complex geometry and bears a great chewing force. The compressive strength test is similar to the chewing behavior. As shown in Figure 13, the compressive strengths of all the hybrid polymer–ceramic materials ranged from 60.36 to 390.46 MPa. The hybrid polymer–ceramic material with 25 wt.% BAG sintered at 1100 °C had the greatest compressive strength. With the increase in the BAG content, the shrinkage rate of the ceramics increased and the compressive strength of the hybrid polymer–ceramic materials also increased.

### 3.10. Biocompatibility

**Figure 14:**
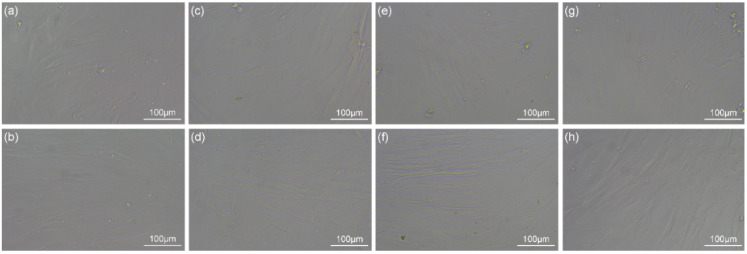
Morphologies of HGFs after 48 h and 72 h culture under 200× light microscope. (**a**,**c**,**e**,**g**) Morphologies of HGFs after 48 h culture; (**b**,**d**,**f**,**h**) Morphologies of HGFs after 72 h culture; (**a**,**b**) control; (**c**,**d**) cured pure resins; (**e**,**f**) HA/BAG ceramics; (**g**,**h**) hybrid polymer–ceramic materials.

**Figure 15:**
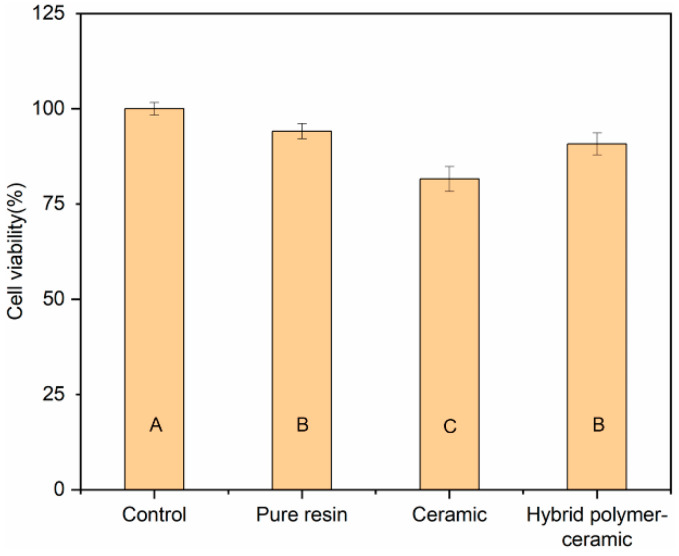
Cell viability of HGFs cultured on different materials for 72 h. ^A^ the same uppercase letters indicate that there was no significant difference between different materials (*p* = 0.05). The data were significantly drawn from a normally distributed population at the 0.05 level.

**Figure 16:**
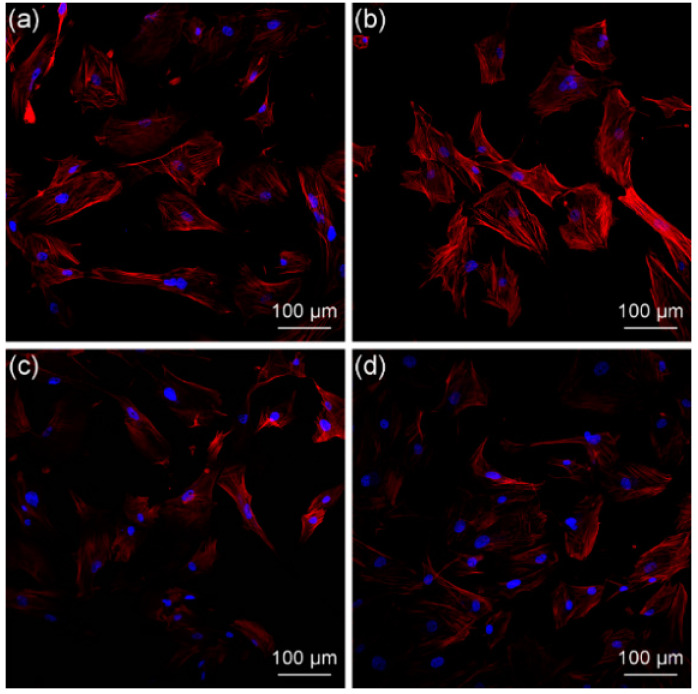
Laser-scanning confocal microscopy images of the cytoskeleton (red; phalloidin) and nuclei (blue; DAPI), showing the HGFs cultured on different materials for 72 h. (**a**) control; (**b**) cured pure resin; (**c**) HA/BAG ceramic; (**d**) hybrid polymer–ceramic material.

The human gingival fibroblasts (HGFs) adhered and proliferated well on the cured pure resin, HA/BAG ceramics, and hybrid polymer–ceramic materials after the 48 h culture. They had a typical elongated fusiform morphology. Over time, the number and coverage of the HGFs after the 72 h culture increased, and the arrangement was compact (Figure 14). No obvious round, floating cells and dead cells were observed. Short-term exposure of HGFs to the polymer did not change the cell morphology. The cell viability of the HGFs after the 72 h culture was measured by the MTT assay (Figure 15), and a statistical difference in the cell proliferation was found between the surfaces of the different materials. The cell viability of the hybrid polymer–ceramic material group was 90.78% of that of control group, which was between the polymer group (94.09%) and ceramic group (81.56%). Figure 16 shows the immunofluorescence staining of HGFs on different materials. The size and morphologies of the nuclei in the four groups were similar. The cytoskeleton of the HGFs in the control group and polymer group was vivid and spread out. In the ceramic group and the hybrid polymer–ceramic material group, the shape of the HGFs was narrow and the cytoskeleton staining was not clear. The growth morphology of HGFs in the ceramic group was the worst.

## 4. Discussion

Enamel and dentin are considered orthotropic due to their microstructural features [27]. In order to achieve a “dental-like” effect, dental restorative materials can recapitulate the biomimetic morphology of tooth tissue [28]. From the micro view (Figure 3 and Figure 4), the ceramic phase and polymer phase of the experimental material are distinct. From a macro view, the ceramic particles and polymers are randomly dispersed, so the material is homogeneous. As in almost all materials used for dental restoration, the hybrid polymer ceramic material is isotropic.

The sintering temperature significantly affects the mechanical properties of hybrid polymer–ceramic materials. An the experiment showed, with the increase in the temperature (1150 °C→1350 °C), the microhardness and flexural strength of the HA/BAG ceramics increased first and then decreased [29]. For hybrid polymer–ceramic materials, the ratio between the ceramic and polymer also influences the mechanical properties. With the increase in the sintering temperature, the shrinkage of the HA/BAG ceramics increases, the porosity decreases, the penetration of monomer mixtures decreases, and the proportion of ceramics in the composites increases. The HA/BAG ceramics sintered at 1000 °C and 1100 °C have interconnected pores that allow for the easy infiltration of the monomer mixtures, which can be used to prepare hybrid polymer–ceramic materials. However, the ceramics sintered at 1200 °C and 1300 °C had glass phases and were dense. This is similar to the experimental results of Chen [19]. The locally closed micropores or dense structure make the monomer mixtures impermeable. So, they are not hybrid polymer–ceramic materials.

The BAG content also influences the mechanical properties of hybrid polymer–ceramic materials. SiO_2_ and Si are key components for the surface activity and biological properties of BAG. They play important roles in the decomposition of HA [30]. The experiment of Li [31] showed that adding a small amount of SiO2 (0.5→1→5 wt.%) promoted the significant conversion of HA to TCP, while adding 5 wt.% and 10 wt.% SiO_2_ had little difference with respect to the amount of HA converted into TCP. Padilla’s experiment confirmed that in 20 wt.% BAG ceramic sintered at 1300 °C, HA was significantly reduced and β-TCP was the main phase [32]. These are similar to the results of our experiment. The hardness, flexural strength, and compressive strength of the hybrid polymer–ceramic material of the pure HA (i.e., 0 wt.% BAG) were all low. With the addition of BAG, the mechanical properties were all improved. This was attributed to the decrease in the porosity and the formation of amorphous glass [33].

For commercial machinable dental materials, the hardness of zirconia ceramics such as Lava TMY-TZP (3M ESPE) is about 13 GPa [34]; the hardness of alumina ceramics (VITA In-Ceram Alumina, Vita Zahnfabrik, Bangkok, Thailand) is about 19.8 GPa; and the hardness of polymethyl methacrylate Esters (Polident PMMA, Polident, Volčja Draga, Slovenia) is about 0.25 GPa [35]. If the material with too high of a hardness and Young’s modulus, such as metal, is used, it will lead to the abrasion of the opposite and adjacent teeth [36,37]. In turn, the lack of contact between the two teeth interferes with the efficiency of chewing [38]. If the hardness of the material is too low, the abrasion resistance and scratch resistance will be poor. Thus, such a material can only be used as a temporary restorative material. The hardness of hybrid polymer–ceramic materials in this experiment is relatively appropriate, which is between enamel (2.7–6.4 GPa) and dentin (0.12–0.67 GPa) (Table 1).

Flexural strength is meaningful for repairing the masticatory function of defective and missing teeth. Especially for fixed bridge restorations or patients with abnormal occlusal habits, the flexural strength of the materials should be as high as possible [39]. The flexural strength of the hybrid polymer–ceramic materials prepared in this experiment matches that of enamel (60–90 MPa) (Table 1). The hybrid polymer–ceramic materials with 20 wt.% BAG sintered at 1000 °C have the best flexural strength. Their hardness, flexural strength, flexural modulus, and compressive strength are 1.26 ± 0.04 GPa, 93.38 ± 7.68 MPa, 23.58 ± 1.51 GPa, and 276.57 ± 52.07 MPa, respectively. Their flexural strength is superior to the machinable dental material VITA CAD-Temp (Vita Zahnfabrik) (80 MPa) [35]. Their flexural modulus is close to the elastic modulus of dentin (8.7–25 GPa). Their compressive strength is close to that of dentin (230–370 MPa) (Table 1). High modulus values are a typical characteristic of stiff materials, such as metal and glass. However, polymers and porous structural materials are generally at low values of this parameter [13]. The flexural modulus of hybrid polymer–ceramic materials sintered at 1100 °C was higher than that sintered at 1000 °C, since high-hardness ceramics were more dominant in the hybrid polymer–ceramic composites. The flexural modulus of the HA/BAG groups at 1300 °C was very large, because the dense ceramics were not easy to deform.

The processing, finishing, and polishing of restorations affects not only the mechanical properties and aesthetics (surface staining and reflectance), but also the health of the soft tissue (plaque accumulation and gingival irritation), and the marginal integrity of the restoration [40]. In this experiment, the same processing and polishing methods were used for different materials, and the surface roughness of the materials was different (Table 2). Using a different finishing technique on the same material also results in inconsistencies in roughness. A rougher surface will increase the adhesion of bacteria, such as *S. sanguinis* [41], one of the bacteria that colonizes the teeth early on, and *Candida albicans* [42], which causes oral infections. Besides roughness, other surface properties (surface charge and surface energy) also influence biofilm formation [43]. There is no further exploration of the material surface properties in this experiment. Therefore, it is necessary to find a more suitable processing and finishing technique for hybrid polymer–ceramic materials.

At present, commercial resin-based CAD/CAM materials contain monomers such as bisphenol-A-glycidyl dimethacrylate (Bis-GMA), triethylene glycol dimethacrylate (TEGDMA), urethane dimethacrylate (UDMA), or bisphenol-A-ethoxylated-glycidyl dimethacrylate (Bis-EMA). In our experiment, Bis-GMA and TEGDMA were selected as organic phases. Bis-GMA has the stable chemical structure of an ether bond, which can prevent the hydrolysis of saliva esterase [44]. The small molecule diluent TEGDMA reduces the viscosity of Bis-GMA. However, Dental materials inevitably interact and cause adverse biological impacts. These include various chemicals in saliva (such as solvolysis, hydrolysis, and alcoholysis) and physical reactions (such as abrasion and erosion) [45]. After exposure to Bis-GMA with a concentration for 50% of the maximal effect (EC50) for 6 h, human gingival fibroblasts (HGFs) were induced to DNA double-strand breaks [46]. TEGDMA also induces mitochondrial damage and oxidative stress in HGFs [47]. Another experiment showed that TEGDMA did not induce DNA strand breaks of HGFs at concentrations up to 10 mM until 24 h [48]. The reason is that the eluted monomers are unlikely to reach the concentrations that may induce necrotic cell death under physiological conditions [49]. According to the data provided by the manufacturers and experiments, the monomers released from commercial resin composite products were below the estimated daily intake limits [50,51,52].

Human gingiva consists of overlying epithelial structures and the underlying connective tissue. Human gingival fibroblasts (HGFs) are the most abundant cells in connective tissue and are very sensitive to the surrounding environment [53]. In addition, HGFs are easy to extract and have strong proliferation. Their culture success rate and resistance were higher than that of human gingival keratinocytes (HGKs) [54]. Studies showed hybrid polymer–ceramic materials were affected HGFs more than HGKs [55,56]. There are also 3D-printed dental restoration and CAD/CAM materials that are used to evaluate biocompatibility through the adhesion and proliferation of HGFs [57,58].

In order to detect the cytotoxicity of our hybrid polymer–ceramic material, the growth of HGFs was tested by the MTT method and immunofluorescence staining. We expected that the HGFs’ morphology was typically spindle-shaped with many long cell processes that explore the environment and communicate with other cells [59]. The results showed that the fusiform HGFs grown on a hybrid polymer–ceramic material presented a well-stretched morphology and a proliferative state. The cell growth of the ceramic group was poor, which was similar to the experimental result of Ruano [60]. In addition to the differences in material types, several studies showed that the surface roughness of the materials influences cell attachment and growth [61]. The surface roughness of the ceramic in this experiment was less than that of the cured pure resin and hybrid polymer–ceramic material, which could explain why the cell viability of HGFs grows in direct contact with the ceramic was lower than that of the other groups. According to the ISO 10993-5:2009 standard, if the cell activity of the samples in the experimental group is more than 70% of that in the blank control group, it is considered to be non-cytotoxic. Therefore, the hybrid polymer–ceramic material has no cytotoxicity and meets the requirements for clinical use. The polymer has no cytotoxic effect on HGFs over a short time.

Nonetheless, the elution of polymer monomers into the oral cavity is a non-linear relationship with time [62]. Monomers can be released in trace amounts for a long time, affecting pulp viability and the prognosis of restored abutment. Furthermore, under the action of enzymes in saliva, monomeric degradation products regulate the growth of *Streptococcus mutans* and *Streptococcus salivarius* [63,64]. Therefore, it is necessary to further study the released elements and the content of monomers to evaluate the long-term safety. In vivo experiments are necessary to demonstrate the effect of an actual clinical application.

## 5. Conclusions

Base on the hybrid polymer–ceramic dental material prepared in this experiment, the following conclusions can be drawn:

(1) The sintering temperature and BAG content affect the mechanical properties of hybrid polymer–ceramic materials;
(2) The mechanical properties of hybrid polymer–ceramic materials are similar to those of natural teeth;
(3) A short-term exposure of human gingival fibroblasts to the hybrid polymer–ceramic material does not cause cytotoxicity.

## Figures and Tables

**Table 1:** The mechanical properties of natural human teeth [20,21,22,23].

	Hardness (GPa)	Flexural Strength (MPa)	Elastic Modulus (GPa)	Compressive Strength (MPa)
Enamel	2.7–6.4	60–90	48–115	95–140
Dentin	0.12–0.67	213–280	8.7–25	230–370

**Table 2:** The surface roughness of different materials.

Material	Sa (nm)	Sq (nm)
Cured pure resin	314.3 ± 186.8	388.3 ± 219.4
Ceramic	237.0 ± 120.1	284.1 ± 141.9
Hybrid polymer–ceramic	256.1 ± 26.6	325.0 ± 31.3

## Data Availability

Not applicable.
